# Stride length and cerebellar regulation: Key features of early gait disorder in cerebral small vessel disease

**DOI:** 10.1111/cns.14545

**Published:** 2024-02-07

**Authors:** Yuting Mo, Biying Ji, Zhihong Ke, Chenglu Mao, Jialiu Jiang, Yanan Huang, Ruomeng Qin, Lili Huang, Dan Yang, Zheqi Hu, Yun Xu

**Affiliations:** ^1^ Department of Neurology, Nanjing Drum Tower Hospital Clinical College of Nanjing Medical University Nanjing China; ^2^ Department of Neurology, Nanjing Drum Tower Hospital Affiliated Hospital of Medical School, Nanjing University Nanjing China; ^3^ Department of Neurology, Nanjing Drum Tower Hospital, State Key Laboratory of Pharmaceutical Biotechnology and Institute of Translational Medicine for Brain Critical Diseases Nanjing University Nanjing China; ^4^ Jiangsu Key Laboratory for Molecular Medicine Medical School of Nanjing University Nanjing China; ^5^ Jiangsu Province Stroke Center for Diagnosis and Therapy Nanjing Drum Tower Hospital Nanjing China

**Keywords:** cerebellum, cerebral small vessel disease, cognitive function, functional connectivity, gait, gray matter

## Abstract

**Objectives:**

Gait disorder (GD) is a common problem in cerebral small vessel disease (CSVD). This study aimed to determine (1) the early characteristics of GD in CSVD, (2) cerebellar neuroimaging features related to GD in CSVD, and (3) the association of cognitive impairment with GD.

**Methods:**

In total, 183 subjects were enrolled in this study: patients with CSVD with normal cognitive function (CSVD‐NC) group (64 subjects), patients with CSVD with mild cognitive impairment (CSVD‐MCI) group (66 subjects), and a healthy control (HC) group (53 subjects). The GD patterns were evaluated using the ReadyGo three‐dimensional motion balance testing system. Meanwhile, we analyzed the cerebrum and cerebellum structurally and functionally. Correlation analyses were conducted among gait indicators, neuroimaging features, and neuropsychological tests.

**Results:**

Both the CSVD‐NC and CSVD‐MCI groups had a reduced stride length, cortical atrophy in the left cerebellum VIIIb, and decreased functional connectivity between the left cerebellum VIIIb and left SFGmed compared with the HC group. In the correlation analysis, the gray matter probability of the left cerebellum VIIIb was closely related to stride length in the HC group. In the CSVD‐MCI group, linguistic function, memory, and attention were significantly correlated with gait performance.

**Conclusion:**

Decreased stride length was the earliest characteristic of GD in CSVD. Structural and functional regulation of the left cerebellum VIIIb could play a particularly important role in early GD in CSVD.

## Graphical abstract

The stride length was reduced in cerebral small vessel disease (CSVD) patients without cognitive impairment and decreased gradually with cognitive decline in CSVD. The structural atrophy and functional down‐regulation of the left cerebellum VIIIb may be responsible for early GD in patients with CSVD.

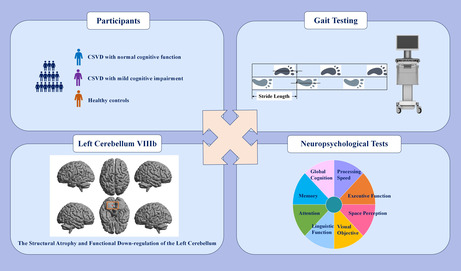

## 1 INTRODUCTION

Cerebral small vessel disease (CSVD), which is a syndrome that displays clinical, neuroimaging, and pathological changes in the intracranial arteriole, capillary, venule, and arteriovenous anastomotic branch,^1^ is prevalent in the elderly population.^2^ Gait disorder (GD) is the second most common problem after cognitive impairment (CI) in CSVD.^3^

GD can bring about an increased risk of falls, frequent accidental injuries, and increased mortality, resulting in a reduction in quality of life and an increased burden on both family and society. In recent years, neuroscientists have paid more attention to cognitive decline rather than GD in CSVD. Nowadays, it is reported that patients with CSVD have poor performance in the timed up and go test, Tinetti test, and gait velocity.^4^ Some researchers believe that GD is closely related to CI in CSVD. For example, patients with CSVD with GD may have an increased risk of dementia.^4^ Similarly, patients with CSVD with gait and balance dysfunction performed worse in cognitive tests than those without.^5^ However, existing studies cannot provide sufficient evidence for a causal or coexisting relationship between GD and CI in patients with CSVD. It was proposed that GD might appear earlier than CI in studies of mild cognitive impairment (MCI) and Alzheimer's disease (AD),^6^, ^7^ while little is known of the changes in gait before CI in patients with CSVD. Therefore, in this study, we grouped patients with CSVD by cognitive grade, so as to concentrate on the relationship between cognition and GD in patients with CSVD.

It is noteworthy that the mechanism underlying GD in CSVD is still unclear. The cerebellum is engaged in sensory motor, coordination, motor control, cognition, and emotional and social psychological function.^8^ In our previous study, patients with CSVD with gait and balance dysfunction displayed cortical atrophy in the bilateral superior temporal gyrus and right cerebellum VIIIa. The structure of the right cerebellum was correlated with the scores on the Tinetti test, but it also showed decreased functional interaction with the cerebrum in patients with CSVD with gait and balance dysfunction.^5^ The cerebellum may deserve more attention in research on the mechanism of GD in patients with CSVD.

Additionally, traditional evaluation methods of GD, such as the Tinetti test, Unified Parkinson's Disease Rating Scale III, timed up and go test, and gait testing, might have insufficient sensitivity for minor gait changes.^9^, ^10^ Owing to limitations in evaluation techniques, most gait testings only include the metric of gait velocity.^4^ The development of artificial intelligence technology provides a more refined way to quantify gait features. The ReadyGo three‐dimensional motion balance testing system, a gait evaluation tool applied with artificial intelligence technology, has shown good performance in fine recognition of gait characteristics.^11^, ^12^

Thus, in this study, we used the ReadyGo three‐dimensional motion balance testing system in the assessment of gait features and utilized neuroimaging techniques aiming to (1) determine the gait characteristics of patients with CSVD with normal cognition (NC) and with MCI, (2) explore the relationship between cognition and gait in patients with CSVD, and (3) examine the mechanism of GD in patients with CSVD using neuroimaging, focusing on the cerebellar structure and its functional interaction with the cerebrum.

## 2 METHODS

### 2.1 Participants

In total, 183 subjects (130 patients with CSVD and 53 healthy controls (HC)) were enrolled in this study at Nanjing Drum Tower Hospital (Nanjing, China) from 2021 to 2023. The study was approved by the Nanjing Drum Tower Hospital Ethics Committee (Ethical Approval Code: 2017‐079‐04) and written informed consent was obtained from each participant. All participants had their background information collected and underwent gait testing, neuropsychological evaluation, and magnetic resonance imaging (MRI) scanning.

The inclusion criteria for the patients with CSVD were (1) age between 40 and 90 years, (2) meeting the diagnostic criteria for CSVD, (3) right‐handed, and (4) cognitive grading as normal or MCI.

The diagnostic criteria for CSVD were as follows: (1) lesions of moderate‐to‐severe white matter hyperintensity (WMH)^13^ (Fazekas scores of 2 or higher) and/or anatomically appropriate lacunes on neuroimaging, with or without enlarged perivascular spaces, microbleeds, and brain atrophy^14^, ^15^; (2) acute symptoms (e.g., lacunar syndromes and transient ischemic attack) or subacute manifestations (e.g., cognitive impairment, motor disturbances, emotional disorders); (3) no history of ischemic stroke with infarct size more than 1.5 cm in diameter; (4) no cerebral hemorrhage; and (5) intracranial or extracranial large artery stenosis ≤50%.

The exclusion criteria for patients with CSVD were as follows: (1) other neurological disorders, such as AD, Parkinson's disease (PD), epilepsy, mental disorder, and so on; (2) any other conditions affecting gait, such as limb weakness, dystonia, joint disease, and so on; (3) serious physical illnesses, such as cancer; (4) limited ability to cooperate with the study protocol, such as blindness, deaf‐mute, physical disability, and so on.

The education‐adjusted cutoff values for the Mini‐Mental State Examination (MMSE) were >17 for illiteracy, >20 for 1–6 years of education, and >24 for >6 years of education. The education‐adjusted cutoff values for the Montreal Cognitive Assessment (MoCA) were >13 for illiteracy, >19 for 1–6 years of education, >24 for 7–12 years of education, and ≥26 for >12 years of education. The NC was defined using both the MoCA^16^ and MMSE scores above the cutoff values adjusted for education. The MCI was defined as (1) cognitive impairment detected by patients, informants, or clinical physicians; (2) having an MMSE score above the cutoff value adjusted for education and a MoCA score below its cutoff value adjusted for education; (3) normal activity of daily living. According to the cognitive grading, patients with CSVD were divided into two groups: patients with CSVD with NC (CSVD‐NC) group (64 subjects) and patients with CSVD with MCI (CSVD‐MCI) group (66 subjects).

The inclusion criteria for the HC group were (1) age between 40 and 90 years, (2) right‐handed, and (3) normal cognitive function. The exclusion criteria for the HC group were as follows: (1) any neurological disorder, such as CSVD, AD, PD, epilepsy, or a mental disorder; (2) any other conditions affecting gait, such as limb weakness, dystonia, or joint disease; (3) serious physical illnesses, such as cancer; and (4) limited ability to cooperate with the study protocol, such as blindness, a deaf‐mute, or physical disability.

### 2.2 Gait testing

The gait testing was performed using the ReadyGo three‐dimensional motion balance testing system.^11^, ^12^ The subjects were asked to walk along a straight path with a flat surface at their preferred speed between two rectangles with a distance of 3 m and a width of 0.5 m for three consecutive rounds and complete the turn within the rectangle. The ReadyGo three‐dimensional motion balance testing system annotated actions such as lifting, landing, turning, standing, and sitting with artificial intelligence, and automatically calculated gait indicators temporally, spatially, and spatiotemporally. The gait indicators included gait velocity, turning time, stride length, step speed, swing velocity, step height, step width, stride frequency, stance phase, swing phase, and double‐support time, with their maximum, minimum, average, median presented. Notably, to improve the accuracy of the gait indicator calculation, the annotated actions were also corrected manually.

### 2.3 Demographic data collection and neuropsychological evaluation

The demographic data collection included sex, age, education, body mass index (BMI), and vascular risk factors. The neuropsychological evaluation contained the Hamilton Depression Rating Scale (HAMD)^17^ and Hamilton Anxiety Rating Scale (HAMA),^18^ MMSE, MoCA,^16^ Digit Span Test (DST), Category Verbal Fluency (CVF), Wechsler Memory Scale‐Visual Reproduction (VR), Stroop Color Word Test (SCWT), Trial Making Test (TMT), Auditory Verbal Learning Test (AVLT),^19^, ^20^ Boston Naming Test (BNT), and Visual Objective and Space Perception Battery—silhouettes (VOSP—silhouettes). Full details are contained in the Appendix S1.

### 2.4 MRI data acquisition

All participants underwent multimodal MRI scanning in the Nanjing Drum Tower Hospital using a Philips 3.0‐T scanner (Philips Medical Systems, the Netherlands). Full details are presented in the Appendix S1.

### 2.5 MRI data analysis

#### 2.5.1 Structural MRI data

Total intracranial volume quantification was conducted using the Voxel‐based morphometry toolbox for statistical parametric mapping software package (SPM; www.fil.ion.ucl.ac.uk/spm) 8. The quantification of WMH was performed using the Lesion Segmentation Tool (LST, a toolbox for SPM12).^21^ FreeSurfer (version 7.2 for Linux)^22^ was used to analyze the difference in cerebral cortical thickness (covariances in Freesurfer: age and total intracranial volume) and structural differences in the cerebellar gray matter were calculated using a spatially unbiased atlas template of the cerebellum and brainstem (SUIT)^23^ (a toolbox based on SPM12) (covariance in SUIT: age). The brain regions that showed significant differences in the structural analyses were used as the focus for the functional analysis.

#### 2.5.2 Functional MRI (fMRI) data

In the fMRI analysis, we focused on functional connectivity (FC). After preprocessing of fMRI data, the brain regions that showed significant differences in the structural analyses were taken as regions of interests (ROIs) to calculate FC using the Data Processing Assistant for Resting‐State Functional MR Imaging toolkit (DPARSF),^24^ adjusted for age.

Full details of the MRI data analysis are contained in the Appendix S1.

### 2.6 Quality control in the MRI analysis

The segmentation and normalization of both structural and functional images were visually inspected and corrected manually if necessary.

### 2.7 Statistical analysis

Demographic data were analyzed using SPSS version 22.0. All continuous variables were tested for normality before comparison by observing whether the data points on the Probability–Probability Plot basically coincide with the diagonal. A chi‐squared test was performed for the comparison of categorical variables. Analysis of variance (ANOVA), general linear model (GLM), and Kruskal–Wallis tests were used to compare variations in continuous variables, in accordance with the results of the test of normality. The statistical analyses for the MRI parameters are described in the Appendix S1. A Spearman's correlation or partial correlation was conducted to explore the relationship among gait indicators, MRI parameters, and neuropsychological data. Covariates used in the GLM and partial correlation that showed nonnormal distribution were log‐transformed. A Bonferroni correction was applied to the ANOVA and GLM results to account for multiple comparisons. All pairwise was chosen for multiple comparisons in the Kruskal–Wallis test. Differences with *p* < 0.05 were considered statistically significant in the analysis of demographic data and gait indicators.

## 3 RESULTS

### 3.1 Demographics, CSVD neuroimaging features, and neuropsychological data

Demographically, there was no significant difference between groups with regard to sex, education, BMI, and vascular risk factors. The age of the CSVD‐NC group was younger than that of the HC and CSVD‐MCI groups. The WMH volume, total lacunes, and total CMBs in the CSVD‐NC and CSVD‐MCI groups were higher than those in the HC group. However, there was no remarkable difference in the cerebellar lacunes and cerebellar CMBs among the three groups. Neuropsychologically, the CSVD‐MCI group performed worse than both the HC and CSVD‐NC groups. Detailed information is shown in Tables 1, 2, 3.

**TABLE 1:** Demographics.

	HC group (*n* = 53)	CSVD‐NC group (*n* = 64)	CSVD‐MCI group (*n* = 66)	Statistics	*p* value
Sex					
Male	23	36	42	4.914	0.086^a^
Female	30	28	24		
Age (years, mean ± SD)	67.264 ± 8.390	65.109 ± 8.547	70.030 ± 6.857	6.292	0.002^b^
Education (years, median [interquartile range])	12.000 (9.000)	12.000 (7.000)	12.000 (5.250)	2.838	0.242^c^
Body mass index (kg/m^2^, mean ± SD)	24.084 ± 2.918	24.531 ± 3.268	24.427 ± 4.253	0.242	0.785^b^
Vascular risk factors					
Hypertension, *n* (%)	28 (52.8%)	37 (52.8%)	39 (59.1%)	0.508	0.776^a^
Diabetes, *n* (%)	10 (18.9%)	18 (28.1%)	19 (28.8%)	1.823	0.402^a^
Hyperlipidemia, *n* (%)	12 (22.6%)	18 (28.1%)	11 (16.7%)	2.456	0.293^a^
Smoking, *n* (%)	9 (17.0%)	10 (15.6%)	18 (27.3%)	3.218	0.200^a^
Drinking, *n* (%)	6 (11.3%)	9 (14.1%)	13 (19.7%)	1.708	0.426^a^

*Note*: A Bonferroni correction was applied to the analysis of variance results to account for multiple comparisons. All pairwise was chosen for multiple comparisons in the Kruskal–Wallis test.

Abbreviations: CSVD‐MCI, patients with cerebral small vessel disease with mild cognitive impairment; CSVD‐NC, patients with cerebral small vessel disease with normal cognitive function; HC, healthy controls.

^a^ Analyzed by chi‐squared test.

^b^ Analyzed by analysis of variance.

^c^ Analyzed by Kruskal–Wallis test.

**TABLE 2:** CSVD neuroimaging features.

	HC group (*n* = 53)	CSVD‐NC group (*n* = 64)	CSVD‐MCI group (*n* = 66)	Statistics	*p* value	Between the HC and CSVD‐NC groups^c^	Between the HC and CSVD‐MCI groups^d^	Between the CSVD‐NC and CSVD‐MCI groups^e^
WMH volume (mL, median [interquartile range])^a^	2.456 (2.027)	8.620 (9.746)	9.522 (13.283)	52.784	0.000	0.000	0.000	1.000
Total lacunes (*n*, median [interquartile range])^a^	0.000 (0.000)	1.000 (2.250)	1.000 (2.000)	61.160	0.000	0.000	0.000	1.000
Cerebellar lacunes (*n*, median [interquartile range])^a^	0.000 (0.000)	0.000 (0.000)	0.000 (0.000)	3.176	0.204			
Total CMBs (*n*, median [interquartile range])^a^	0.000 (1.000)	1.000 (2.000)	1.000 (2.000)	6.750	0.034	0.103	0.177	1.000
Cerebellar CMBs (*n*, median [interquartile range])^a^	0.000 (0.000)	0.000 (0.000)	0.000 (0.000)	5.293	0.071			
Total intracranial volume (mL, mean ± SD)^b^	1362.768 ± 130.921	1406.770 ± 117.784	1373.945 ± 129.802	1.987	0.140			

*Note*: All pairwise was chosen for multiple comparisons in the Kruskal–Wallis test.

Abbreviations: CMBs, cerebral microbleeds; CSVD‐MCI, patients with cerebral small vessel disease with mild cognitive impairment; CSVD‐NC, patients with cerebral small vessel disease with normal cognitive function; HC, healthy controls; WMH, white matter hyperintensity.

^a^ Analyzed by Kruskal–Wallis test.

^b^ Analyzed by analysis of variance.

^c^ *p* value between the HC and CSVD‐NC groups.

^d^ *p* value between the HC and CSVD‐MCI groups.

^e^ *p* value between the CSVD‐NC and CSVD‐MCI groups.

**TABLE 3:** Neuropsychological data.

	HC group (*n* = 53)	CSVD‐NC group (*n* = 64)	CSVD‐MCI group (*n* = 66)	Statistics	*p* value	Observed Power^c^	Between the HC and CSVD‐NC groups^d^	Between the HC and CSVD‐MCI groups^e^	Between the CSVD – NC and CSVD‐MCI groups^f^
HAMD (median [interquartile range])^a^	4.000 (5.750)	4.000 (5.000)	4.000 (6.000)	1.103	0.576				
HAMA (median [interquartile range])^a^	5.000 (5.000)	7.000 (6.000)	6.000 (7.000)	5.655	0.059				
MMSE (median [interquartile range])^a^	29.000 (2.000)	29.000 (1.750)	28.000 (3.000)	24.658	0.000		0.633	0.000	0.003
MoCA (median [interquartile range])^a^	26.000 (2.500)	27.000 (3.000)	21.000 (6.000)	85.819	0.000		0.837	0.000	0.000
DSF (median [interquartile range])^a^	8.000 (2.250)	9.000 (1.000)	8.000 (2.000)	5.213	0.074				
DSB (median [interquartile range])^a^	4.500 (2.000)	5.000 (1.500)	4.000 (2.000)	11.181	0.004		1.000	0.007	0.020
CVF (mean ± SD)^b^	18.313 ± 4.830	18.596 ± 5.209	14.538 ± 4.390	9.890	0.000	0.171	1.000	0.001	0.000
VRIR (median [interquartile range])^a^	10.000 (3.750)	10.000 (4.000)	7.000 (4.000)	23.524	0.000		1.000	0.000	0.001
VRDR (median [interquartile range])^a^	8.000 (5.000)	9.000 (6.000)	4.000 (7.000)	28.384	0.000		1.000	0.000	0.000
VRR (median [interquartile range])^a^	2.000 (1.000)	3.000 (2.000)	1.000 (2.000)	16.963	0.000		0.630	0.000	0.026
SCWT‐dot (median [interquartile range])^a^	18.000 (6.000)	18.000 (8.000)	21.500 (15.675)	12.709	0.002		1.000	0.002	0.020
SCWT‐dot (error) (median [interquartile range])^a^	0.000 (0.000)	0.000 (0.000)	0.000 (0.000)	11.964	0.003		1.000	0.004	0.024
SCWT‐word (median [interquartile range])^a^	20.000 (7.000)	20.500 (9.000)	27.000 (13.500)	21.709	0.000		1.000	0.000	0.000
SCWT‐word (error) (median [interquartile range])^a^	0.000 (0.000)	0.000 (0.000)	0.000 (0.000)	5.331	0.070				
SCWT‐interference (median [interquartile range])^a^	27.000 (15.000)	28.000 (13.000)	35.500 (22.250)	12.226	0.002		1.000	0.003	0.030
SCWT‐interference (error) (median [interquartile range])^a^	1.000 (2.000)	0.000 (1.000)	1.500 (2.000)	17.534	0.000		0.520	0.000	0.027
SCWT‐interference‐dot (median [interquartile range])^a^	8.000 (15.500)	9.000 (11.000)	13.000 (16.500)	2.030	0.362				
SCWT‐interference‐word (median [interquartile range])^a^	7.000 (14.750)	7.000 (10.000)	9.500 (13.000)	0.685	0.710				
TMT‐A (median [interquartile range])^a^	58.500 (33.000)	55.000 (24.000)	81.000 (52.000)	21.843	0.000		0.336	0.000	0.015
TMT‐B (median [interquartile range])^a^	110.000 (75.750)	91.000 (76.500)	160.000 (134.250)	19.768	0.000		1.000	0.000	0.003
TMT‐BA (median [interquartile range])^a^	46.000 (60.750)	38.000 (47.500)	79.000 (126.875)	12.399	0.002		1.000	0.002	0.032
AVLTIR (mean ± SD)^b^	16.546 ± 0.684	16.260 ± 0.650	12.813 ± 0.656	9.707	0.000	0.784	1.000	0.000	0.001
AVLTSTDR (median [interquartile range])^a^	5.000 (3.000)	5.000 (3.000)	3.000 (3.000)	26.766	0.000		1.000	0.000	0.000
AVLTLTDR (median [interquartile range])^a^	5.000 (2.000)	5.000 (3.000)	2.000 (5.000)	31.770	0.000		1.000	0.000	0.000
AVLT‐recognition (median [interquartile range])^a^	21.000 (4.000)	22.000 (3.000)	20.000 (4.000)	20.030	0.000		1.000	0.000	0.003
BNT (median [interquartile range])^a^	52.000 (11.000)	53.000 (8.500)	46.000 (12.000)	22.129	0.000		0.757	0.010	0.249
VOSP‐silhouettes (mean ± SD)^b^	9.332 ± 0.423	10.040 ± 0.412	8.315 ± 0.404	4.411	0.014	0.087	0.695	0.254	0.011

*Note*: A Bonferroni correction was applied to the general linear model results to account for multiple comparisons. All pairwise was chosen for multiple comparisons in the Kruskal–Wallis test.

Abbreviations: AVLTIR: Auditory Verbal Learning Test‐immediate recall; AVLTLTDR: Auditory Verbal Learning Test‐long‐time delay recall; AVLT‐recognition: Auditory Verbal Learning Test‐recognition; AVLTSTDR: Auditory Verbal Learning Test‐short‐time delay recall; BNT, Boston Naming Test; CSVD‐MCI, patients with cerebral small vessel disease with mild cognitive impairment; CSVD‐NC, patients with cerebral small vessel disease with normal cognitive function; CVF, Category Verbal Fluency; DSB, Digit Span Test‐backward; DSF, Digit Span Test‐forward; HAMA, Hamilton Anxiety Rating Scale; HAMD, Hamilton Depression Rating Scale; HC, healthy controls; MMSE, MiniMental State Examination; MoCA, Montreal Cognitive Assessment; SCWT, Stroop Color Word Test; TMT, Trial Making Test; VOSP‐silhouettes, Visual Objective and Space Perception Battery‐silhouettes; VRDR, Visual Reproduction‐Delayed Recall; VRIR, Visual Reproduction‐Immediate Recall; VRR, Visual Reproduction‐Recognition.

^a^ Analyzed by Kruskal–Wallis test.

^b^ Analyzed by general linear model, adjusted for age.

^c^ Observed Power of age in Tests of Between‐Subjects Effects in general linear model.

^d^ *p* value between the HC and CSVD‐NC groups.

^e^ *p*‐value between the HC and CSVD‐MCI groups.

^f^ *p*‐value between the CSVD‐NC and CSVD‐MCI groups.

### 3.2 Gait indicators

**FIGURE 1:**
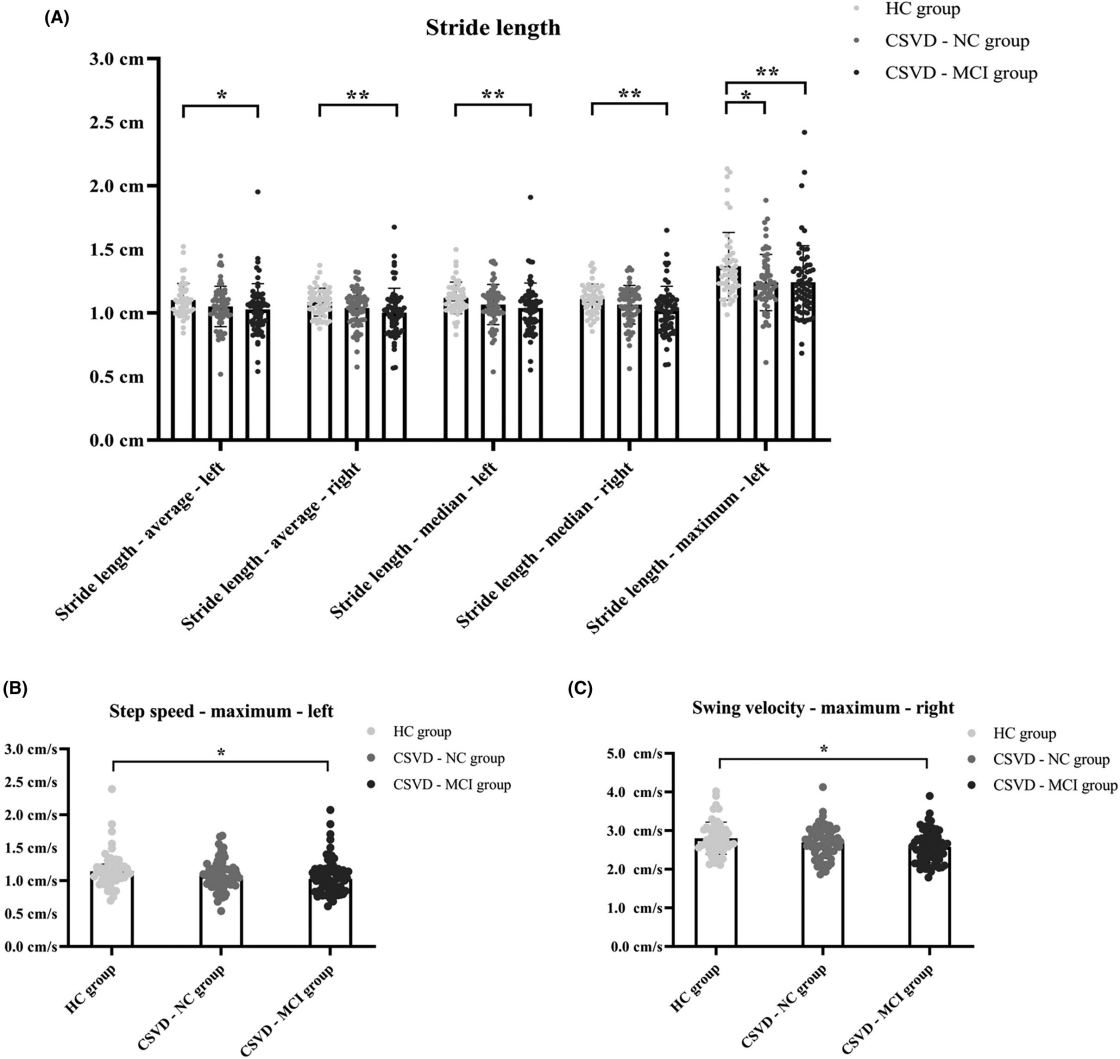
(A–C) Gait indicators with significant differences among the HC, CSVD‐NC, and CSVD‐MCI groups. The detailed statistics are shown in the Appendix S1. CSVD‐MCI, patients with cerebral small vessel disease with mild cognitive impairment; CSVD‐NC, patients with cerebral small vessel disease with normal cognitive function; HC, healthy controls. *p < 0.05; **p < 0.01.

A GLM was applied in the comparison of gait indicators conforming to a normal distribution (adjusted for age) and a Kruskal–Wallis test was used in the analysis of gait indicators that showed a skewed distribution among the HC, CSVD‐NC, and CSVD‐MCI groups. The HC group showed better performance in stride length‐average‐left, stride length‐average‐right, stride length‐median‐left, stride length‐median‐right, stride length‐maximum‐left, step speed‐maximum‐left, and swing velocity‐maximum‐right than the CSVD‐MCI group. Notably, the HC group also performed a larger stride length‐maximum‐left than the CSVD‐NC group. Detailed information is shown in Figure 1 and Table 4.

**TABLE 4:** Gait indicators.

	HC group (*n* = 53)	CSVD‐NC group (*n* = 64)	CSVD‐MCI group (*n* = 66)	Statistics	*p* value	Observed Power^c^	Between the HC and CSVD‐NC groups^d^	Between the HC and CSVD‐MCI groups^e^	Between the CSVD‐NC and CSVD‐MCI groups^f^
Gait velocity (cm/s, mean ± SD)^b^	0.874 ± 0.136	0.860 ± 0.148	0.820 ± 0.167	1.167	0.314	0.891			
Turning time (s, median [interquartile range])^a^	1.283 (0.350)	1.400 (0.413)	1.442 (0.534)	4.304	0.116				
Stride length‐average‐left (cm, median [interquartile range])^a^	1.085 (0.169)	1.049 (0.165)	1.038 (0.212)	8.364	0.015		0.278	0.012	0.626
Stride length‐average‐right (cm, median [interquartile range])^a^	1.093 (0.154)	1.056 (0.185)	1.006 (0.233)	12.425	0.002		0.296	0.001	0.157
Stride length‐median‐left (cm, median [interquartile range])^a^	1.115 (0.153)	1.074 (0.148)	1.049 (0.209)	9.595	0.008		0.194	0.006	0.585
Stride length‐median‐right (cm, median [interquartile range])^a^	1.109 (0.167)	1.100 (0.164)	1.024 (0.224)	13.173	0.001		0.525	0.001	0.064
Stride length‐maximum‐left (cm, median [interquartile range])^a^	1.300 (0.224)	1.213 (0.240)	1.203 (0.299)	10.104	0.006		0.029	0.009	1.000
Stride length‐maximum‐right (cm, mean ± SD)^b^	1.318 ± 0.186	1.244 ± 0.200	1.230 ± 0.274	2.483	0.086	0.879			
Stride length‐minimum‐left (cm, mean ± SD)^b^	0.811 ± 0.192	0.828 ± 0.193	0.782 ± 0.215	0.628	0.535	0.102			
Stride length‐minimum‐right (cm, mean ± SD)^b^	0.813 ± 0.176	0.790 ± 0.190	0.744 ± 0.219	1.721	0.182	0.060			
Step speed‐average‐left (cm/s, mean ± SD)^b^	0.944 ± 0.150	0.912 ± 0.168	0.880 ± 0.196	1.482	0.230	0.805			
Step speed‐average‐right (cm/s, mean ± SD)^b^	0.929 ± 0.139	0.905 ± 0.162	0.865 ± 0.184	1.548	0.216	0.683			
Step speed‐median‐left (cm/s, mean ± SD)^b^	0.960 ± 0.143	0.923 ± 0.166	0.890 ± 0.192	1.954	0.145	0.781			
Step speed‐median‐right (cm/s, mean ± SD)^b^	0.946 ± 0.147	0.922 ± 0.164	0.879 ± 0.186	1.621	0.201	0.739			
Step speed‐maximum‐left (cm/s, median [interquartile range])^a^	1.142 (0.215)	1.074 (0.243)	1.026 (0.316)	8.911	0.012		0.245	0.009	0.588
Step speed‐maximum‐right (cm/s, mean ± SD)^b^	1.121 ± 0.169	1.099 ± 0.219	1.067 ± 0.254	0.525	0.593	0.886			
Step speed‐minimum‐left (cm/s, mean ± SD)^b^	0.699 ± 0.182	0.696 ± 0.166	0.661 ± 0.190	0.639	0.529	0.113			
Step speed‐minimum‐right (cm/s, median [interquartile range])^a^	0.687 (0.232)	0.673 (0.289)	0.613 (0.247)	3.249	0.197				
Swing velocity‐average‐left (cm/s, mean ± SD)^b^	2.096 ± 0.256	2.108 ± 0.319	2.026 ± 0.357	0.691	0.502	0.327			
Swing velocity‐average‐right (cm/s, mean ± SD)^b^	2.137 ± 0.296	2.137 ± 0.308	2.019 ± 0.339	2.091	0.127	0.293			
Swing velocity‐median‐left (cm/s, mean ± SD)^b^	2.208 ± 0.268	2.174 ± 0.314	2.101 ± 0.367	1.207	0.302	0.441			
Swing velocity‐median‐right (cm/s, mean ± SD)^b^	2.216 ± 0.307	2.206 ± 0.314	2.095 ± 0.364	1.684	0.189	0.415			
Swing velocity‐maximum‐left (cm/s, mean ± SD)^b^	2.742 ± 0.370	2.673 ± 0.398	2.598 ± 0.456	1.368	0.257	0.480			
Swing velocity‐maximum‐right (cm/s, mean ± SD)^b^	2.803 ± 0.418	2.693 ± 0.413	2.577 ± 0.411	3.580	0.030	0.563	0.293	0.026	0.936
Swing velocity‐minimum‐left (cm/s, mean ± SD)^b^	1.233 ± 0.412	1.335 ± 0.472	1.234 ± 0.445	1.026	0.361	0.050			
Swing velocity‐minimum‐right (cm/s, mean ± SD)^b^	1.297 ± 0.432	1.385 ± 0.373	1.249 ± 0.419	2.216	0.112	0.160			
Stride frequency‐average‐left (steps/min, mean ± SD)^b^	103.064 ± 11.344	105.462 ± 10.225	103.931 ± 11.675	0.623	0.538	0.070			
Stride frequency‐average‐right (steps/min, mean ± SD)^b^	103.217 ± 10.810	103.472 ± 10.469	103.113 ± 11.178	0.028	0.972	0.055			
Stride frequency‐median‐left (steps/min, median [interquartile range])^a^	100.000 (16.109)	105.882 (14.474)	105.882 (17.763)	1.162	0.559				
Stride frequency‐median‐right (steps/min, median [interquartile range])^a^	100.000 (17.763)	105.882 (17.763)	100.000 (17.763)	0.264	0.876				
Stride frequency‐maximum‐left (steps/min, median [interquartile range])^a^	112.500 (22.689)	120.000 (22.689)	120.000 (22.689)	1.511	0.470				
Stride frequency‐maximum‐right (steps/min, median [interquartile range])^a^	112.500 (22.689)	120.000 (22.689)	112.500 (22.689)	0.754	0.686				
Stride frequency‐minimum‐left (steps/min, median [interquartile range])^a^	90.000 (18.182)	90.000 (9.023)	90.000 (18.182)	1.624	0.444				
Stride frequency‐minimum‐right (steps/min, median [interquartile range])^a^	90.000 (14.286)	90.000 (12.919)	90.000 (12.919)	0.870	0.647				
Stance phase‐average‐left (s, mean ± SD)^b^	67.674 ± 1.891	67.817 ± 1.898	68.071 ± 2.072	0.330	0.720	0.882			
Stance phase‐average‐right (s, mean ± SD)^b^	67.686 ± 1.967	68.153 ± 1.753	67.898 ± 1.958	1.275	0.282	0.399			
Stance phase – median – left (s, median [interquartile range])^a^	67.647 (1.986)	67.647 (1.904)	67.836 (2.430)	1.890	0.389				
Stance phase‐median‐right (s, mean ± s.d.)^b^	67.549 ± 1.797	67.843 ± 1.497	67.833 ± 2.048	0.526	0.592	0.245			
Stance phase‐maximum‐left (s, median [interquartile range])^a^	71.795 (3.111)	72.222 (3.804)	72.047 (3.940)	0.494	0.781				
Stance phase‐maximum‐right (s, median [interquartile range])^a^	71.429 (4.625)	72.457 (4.412)	72.049 (2.980)	1.219	0.544				
Stance phase‐minimum‐left (s, median [interquartile range])^a^	63.889 (3.264)	63.636 (2.292)	64.167 (4.143)	1.032	0.597				
Stance phase‐minimum‐right (s, median [interquartile range])^a^	63.636 (3.213)	63.889 (2.365)	64.286 (3.556)	1.604	0.448				
Swing phase‐average‐left (s, mean ± SD)^b^	32.326 ± 1.891	32.183 ± 1.898	31.929 ± 2.072	0.330	0.720	0.882			
Swing phase‐average‐right (s, mean ± SD)^b^	32.314 ± 1.967	31.847 ± 1.753	32.102 ± 1.958	1.275	0.282	0.399			
Swing phase‐median‐left (s, median [interquartile range])^a^	32.353 (1.986)	32.353 (1.904)	32.164 (2.430)	1.890	0.389				
Swing phase‐median‐right (s, mean ± SD)^b^	32.451 ± 1.797	32.157 ± 1.497	32.167 ± 2.048	0.526	0.592	0.245			
Swing phase‐maximum‐left (s, median [interquartile range])^a^	36.111 (3.264)	36.364 (2.292)	35.833 (4.143)	1.032	0.597				
Swing phase‐maximum‐right (s, median [interquartile range])^a^	36.364 (3.213)	36.111 (2.365)	35.714 (3.556)	1.604	0.448				
Swing phase‐minimum‐left (s, median [interquartile range])^a^	28.205 (3.111)	27.778 (3.804)	27.953 (3.941)	0.494	0.781				
Swing phase‐minimum‐right (s, median [interquartile range])^a^	28.571 (4.625)	27.543 (4.412)	27.951 (2.980)	1.219	0.544				
Double‐support time‐average‐left (s, mean ± SD)^b^	34.806 ± 2.948	35.078 ± 2.855	35.551 ± 3.616	0.454	0.636	0.859			
Double‐support time‐average‐right (s, mean ± SD)^b^	34.806 ± 2.948	35.078 ± 2.855	35.551 ± 3.616	0.454	0.636	0.859			
Double‐support time‐median‐left (s, mean ± SD)^b^	34.794 ± 3.029	35.019 ± 2.817	35.467 ± 3.603	0.347	0.707	0.839			
Double‐support time‐median‐right (s, mean ± SD)^b^	34.794 ± 3.029	35.019 ± 2.817	35.467 ± 3.603	0.347	0.707	0.839			
Double‐support time‐maximum‐left (s, mean ± SD)^b^	40.429 ± 3.405	40.924 ± 3.466	41.717 ± 4.353	1.112	0.331	0.803			
Double‐support time‐maximum‐right (s, mean ± SD)^b^	40.429 ± 3.405	40.924 ± 3.466	41.717 ± 4.353	1.112	0.331	0.803			
Double‐support time‐minimum‐left (s, median [interquartile range])^a^	29.545 (4.156)	29.222 (3.761)	30.369 (5.394)	1.247	0.536				
Double‐support time‐minimum‐right (s, median [interquartile range])^a^	29.545 (4.156)	29.222 (3.761)	30.369 (5.394)	1.247	0.536				
Step height‐average‐left (cm, mean ± SD)^b^	0.126 ± 0.021	0.125 ± 0.021	0.122 ± 0.019	0.338	0.714	0.222			
Step height‐average‐right (cm, mean ± SD)^b^	0.121 ± 0.018	0.118 ± 0.024	0.117 ± 0.021	0.511	0.601	0.264			
Step height‐median‐left (cm, mean ± SD)^b^	0.125 ± 0.023	0.124 ± 0.023	0.122 ± 0.020	0.246	0.783	0.050			
Step height‐median‐right (cm, mean ± SD)^b^	0.120 ± 0.019	0.115 ± 0.022	0.116 ± 0.023	1.012	0.366	0.085			
Step height‐maximum‐left (cm, mean ± SD)^b^	0.190 ± 0.035	0.186 ± 0.036	0.181 ± 0.028	0.771	0.464	0.421			
Step height‐maximum‐right (cm, median [interquartile range])^a^	0.180 (0.053)	0.177 (0.043)	0.181 (0.046)	0.950	0.622				
Step height‐minimum‐left (cm, mean ± SD)^b^	0.055 ± 0.027	0.061 ± 0.026	0.055 ± 0.026	0.883	0.415	0.975			
Step height‐minimum‐right (cm, mean ± SD)^b^	0.054 ± 0.020	0.052 ± 0.022	0.052 ± 0.021	0.128	0.880	0.740			
Step width‐average (cm, mean ± SD)^b^	0.128 ± 0.018	0.134 ± 0.021	0.134 ± 0.021	1.621	0.201	1.000			
Step width‐median (cm, median [interquartile range])^a^	0.126 (0.024)	0.133 (0.031)	0.131 (0.030)	3.384	0.184				
Step width‐maximum (cm, median [interquartile range])^a^	0.144 (0.026)	0.153 (0.033)	0.147 (0.041)	4.448	0.108				
Step width‐minimum (cm, median [interquartile range])^a^	0.112 (0.030)	0.113 (0.029)	0.114 (0.029)	0.237	0.888				

*Note*: A Bonferroni correction was applied to the analysis of variance results to account for multiple comparisons. All pairwise was chosen for multiple comparisons in the Kruskal–Wallis test.

Abbreviations: CSVD‐MCI, patients with cerebral small vessel disease with mild cognitive impairment; CSVD‐NC, patients with cerebral small vessel disease with normal cognitive function; HC, healthy controls.

^a^ Analyzed by Kruskal–Wallis test.

^b^ Analyzed by a general linear model, adjusted for age.

^c^ Observed Power of age in Tests of Between‐Subjects Effects in a general linear model.

^d^ *p* value between the HC and CSVD‐NC groups.

^e^ *p* value between the HC and CSVD‐MCI groups.

^f^ *p* value between the CSVD‐NC and CSVD‐MCI groups.

### 3.3 Structural analysis

**FIGURE 2:**
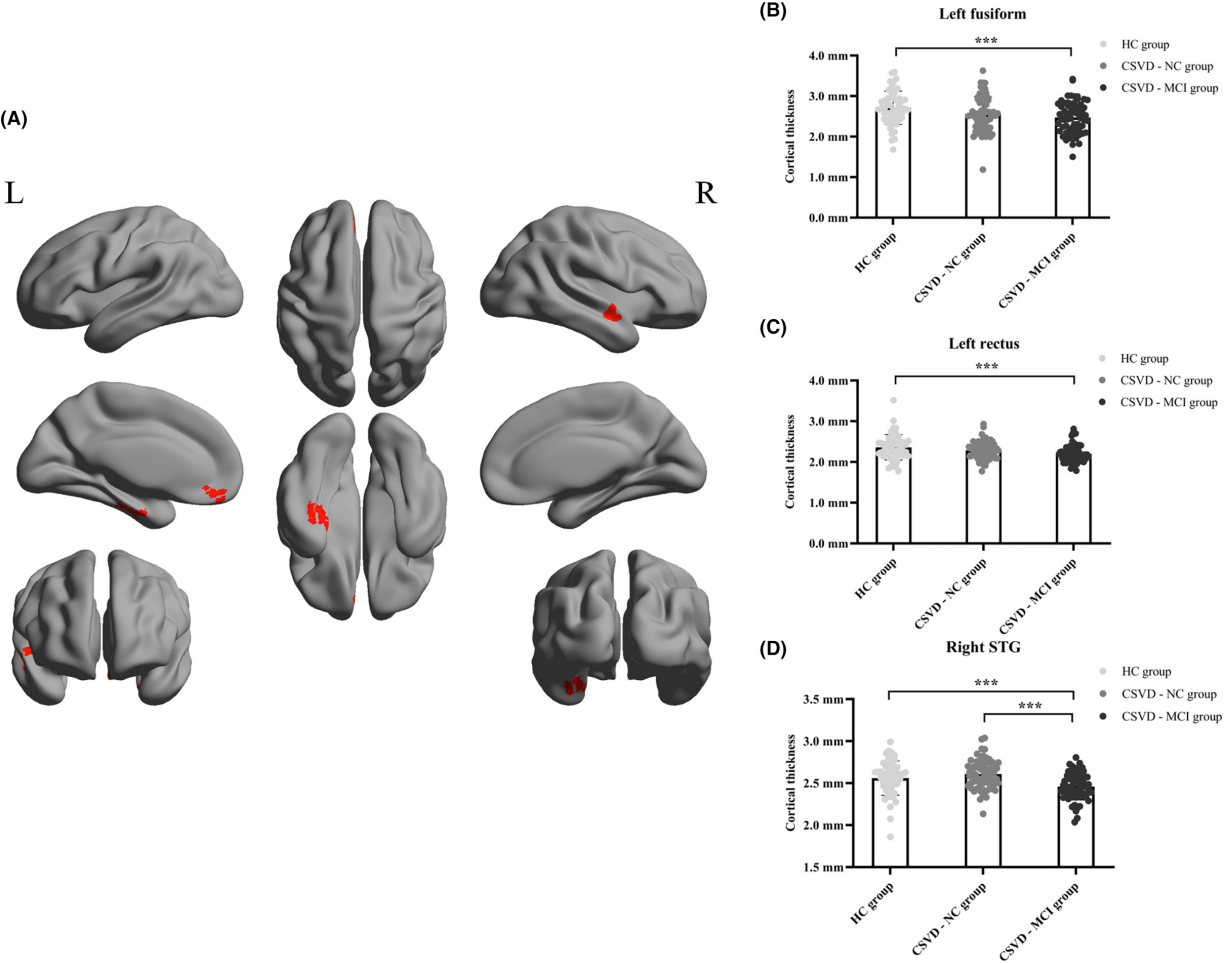
Brain regions with significant differences in cerebral cortical thickness among the HC, CSVD‐NC, and CSVD‐MCI groups (Monte Carlo simulation correction for multiple comparisons, vertex‐wise/cluster‐forming: *p* < 0.001, cluster‐wise: *p* < 0.05). (A) The red‐shaded areas correspond to the brain regions with significant difference in cerebral cortical thickness: the left fusiform (Montreal Neurological Institute coordinates of peak vertex: *x* = −30.0, *y* = −22.0, *z* = −27.5, cluster size: 281.22 mm^2^), left rectus (Montreal Neurological Institute coordinates of peak vertex: *x* = −4.2, *y* = 41.7, *z* = −23.7, cluster size: 137.17 mm^2^), and right STG (Montreal Neurological Institute coordinates of peak vertex: *x* = −55.2, *y* = −4.5, *z* = −3.8, cluster size: 386.40 mm^2^). (B) The cortical thickness of the left fusiform in the HC group (2.785 ± 0.392 mm), CSVD‐NC group (2.585 ± 0.398 mm), and CSVD‐MCI group (2.380 ± 0.374 mm). (C) The cortical thickness of the left rectus in the HC group (2.404 ± 0.284 mm), CSVD‐NC group (2.286 ± 0.208 mm), and CSVD‐MCI group (2.162 ± 0.218 mm). (D) The cortical thickness of the right STG in the HC group (2.567 ± 0.189 mm), CSVD‐NC group (2.628 ± 0.155 mm), and CSVD‐MCI group (2.433 ± 0.165 mm). CSVD‐MCI, patients with cerebral small vessel disease with mild cognitive impairment; CSVD‐NC, patients with cerebral small vessel disease with normal cognitive function; HC, healthy controls; STG: superior temporal gyrus. ***p < 0.001.

With regard to the cerebrum, the cortical thickness in the left fusiform, left rectus, and right STG (superior temporal gyrus) were thinner in the CSVD‐MCI group than in the HC group. The cortical atrophy of the right STG also existed in the CSVD‐MCI group than in the CSVD‐NC group. However, there was no remarkable difference in cerebral cortical thickness between the HC and CSVD‐NC groups. Monte Carlo simulation correction was used for multiple comparisons in FreeSurfer (vertex‐wise/cluster‐forming: *p* < 0.001, cluster‐wise: *p* < 0.05) (Figure 2).

**FIGURE 3:**
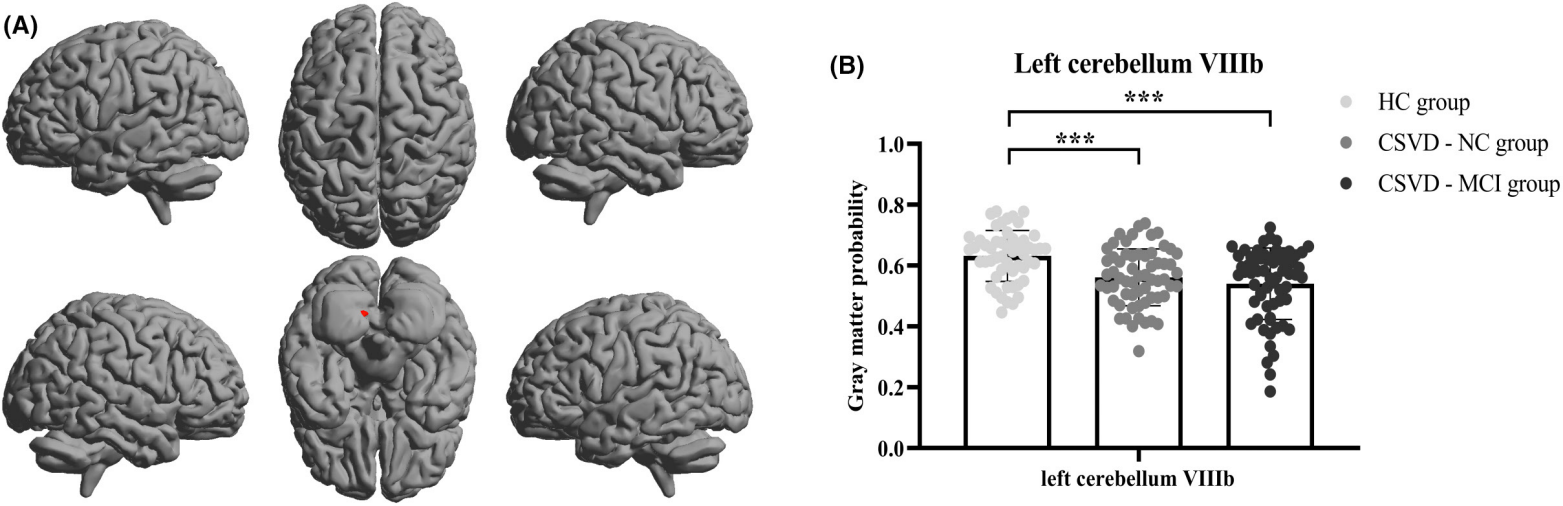
The brain region with significant differences in cerebellar gray matter probability among the HC, CSVD‐NC, and CSVD‐MCI groups (Gaussian random field correction for multiple comparisons, voxel level: *p* < 0.001, cluster level: *p* < 0.05). (A) The red‐shaded area corresponds to the left cerebellum VIIIb (cerebellum_8_L, Montreal Neurological Institute coordinates of peak voxel: *x* = −9, *y* = −64, *z* = −44, cluster size: 136 voxels) which showed a significant difference in cerebellar gray matter probability among the HC, CSVD‐NC, and CSVD‐MCI groups. (B) The gray matter probability of the left cerebellum VIIIb in the HC group (0.632 ± 0.084), CSVD‐NC group (0.562 ± 0.093), and CSVD‐MCI group (0.540 ± 0.117). CSVD‐MCI, patients with cerebral small vessel disease with mild cognitive impairment; CSVD‐NC, patients with cerebral small vessel disease with normal cognitive function; HC, healthy controls. ***p < 0.001.

With regard to the cerebellum, six subjects (one in the HC group, three in the CSVD‐NC group, and two in the CSVD‐MCI group) were excluded owing to unsatisfactory segmentation. The gray matter probability of the left cerebellum VIIIb in the HC group was higher than in both the CSVD‐NC and CSVD‐MCI groups (Gaussian random field correction [GRF] for multiple comparisons, voxel level: *p* < 0.001, cluster level: *p* < 0.05; Figure 3).

### 3.4 FC analysis

The FC calculation concentrated on regions that showed significant differences in the structural analysis. The MNI coordinates of the peak vertex in the left fusiform, left rectus, right STG, as well as the MNI coordinate of peak voxel in the left cerebellum VIIIb were defined as the spherical center of ROIs (5‐mm radius spheres) in the FC analysis. Notably, only 50 subjects in the HC group, 63 subjects in the CSVD‐NC group, and 59 subjects in the CSVD‐MCI group were included in the FC analysis. Eleven subjects (three in the HC group, one in the CSVD‐NC group, and seven in the CSVD‐MCI group) were excluded owing to unqualified head motion in the functional image.

**FIGURE 4:**
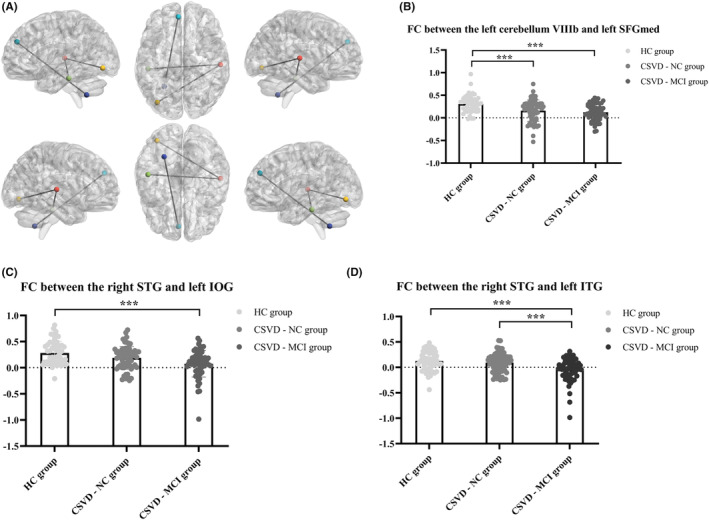
Significant FC among the HC, CSVD‐NC, and CSVD‐MCI groups (adjusted for age, Gaussian random field correction for multiple comparisons, voxel level: *p* < 0.001, cluster level: *p* < 0.05). (A) The colored spheres correspond to brain regions in FC analysis. The sticks represent significant FC between brain regions. The dark blue sphere is the left cerebellum VIIIb (regions of interest in FC analysis, MNI coordinates of the peak voxel: *x* = −9, *y* = −64, *z* = −44). The red sphere is the right STG (regions of interest in FC analysis, MNI coordinates of the peak voxel: *x* = −55.2, *y* = −4.5, *z* = −3.8). The baby blue sphere is the left SFGmed (MNI coordinates of the peak voxel: *x* = 0, *y* = 42, *z* = 30, cluster size: 37 voxels). The yellow sphere is the left IOG (MNI coordinates of the peak voxel: *x* = −39, *y* = −93, *z* = −15, cluster size: 39 voxels). The green sphere is the left ITG (MNI coordinates of the peak voxel: *x* = −39, *y* = −18, *z* = −36, cluster size: 40 voxels). (B) FC between the left cerebellum VIIIb and left SFGmed among the HC (mean ± SD: 0.307 ± 0.189), CSVD‐NC (mean ± SD: 0.158 ± 0.239), and CSVD‐MCI (mean ± SD: 0.124 ± 0.182) groups. (C) FC between the right STG and left IOG among the HC (mean ± SD: 0.283 ± 0.226), CSVD‐NC (mean ± SD: 0.189 ± 0.219), and CSVD‐MCI (mean ± SD: 0.077 ± 0.255) groups. (D) FC between the right STG and left ITG among the HC (mean ± SD: 0.123 ± 0.190), CSVD‐NC (mean ± SD: 0.093 ± 0.187), and CSVD‐MCI (mean ± SD: −0.040 ± 0.226) groups. CSVD‐MCI, patients with cerebral small vessel disease with mild cognitive impairment; CSVD‐NC, patients with cerebral small vessel disease with normal cognitive function; FC, functional connectivity; HC, healthy controls; IOG, inferior occipital gyrus; ITG, inferior temporal gyrus; MNI, Montreal Neurological Institute; SFGmed, superior frontal gyrus, medial; STG, superior temporal gyrus. ***p < 0.001.

Both the left fusiform and left rectus showed no remarkable difference among the three groups in the FC analysis. The CSVD‐MCI group had lower FC than the HC group between the right STG and left IOG (inferior occipital gyrus) (GRF for multiple comparisons, voxel level: *p* < 0.001, cluster level: *p* < 0.05). The CSVD‐MCI group also showed lower FC than the HC and CSVD‐NC groups between the right STG and the left ITG (inferior temporal gyrus) (GRF for multiple comparisons, voxel level: *p* < 0.001, cluster level: *p* < 0.05). However, no difference was observed between the HC and CSVD‐NC groups. (Figure 4).

Notably, the FC between the left cerebellum VIIIb and left SFGmed (superior frontal gyrus, medial) was significantly reduced in the CSVD‐NC and CSVD‐MCI groups compared with the HC group, while no remarkable reduction was found between the CSVD‐NC and CSVD‐MCI groups (GRF for multiple comparisons, voxel level: *p* < 0.001, cluster level: *p* < 0.05). (Figure 4).

### 3.5 Correlation analysis

**FIGURE 5:**
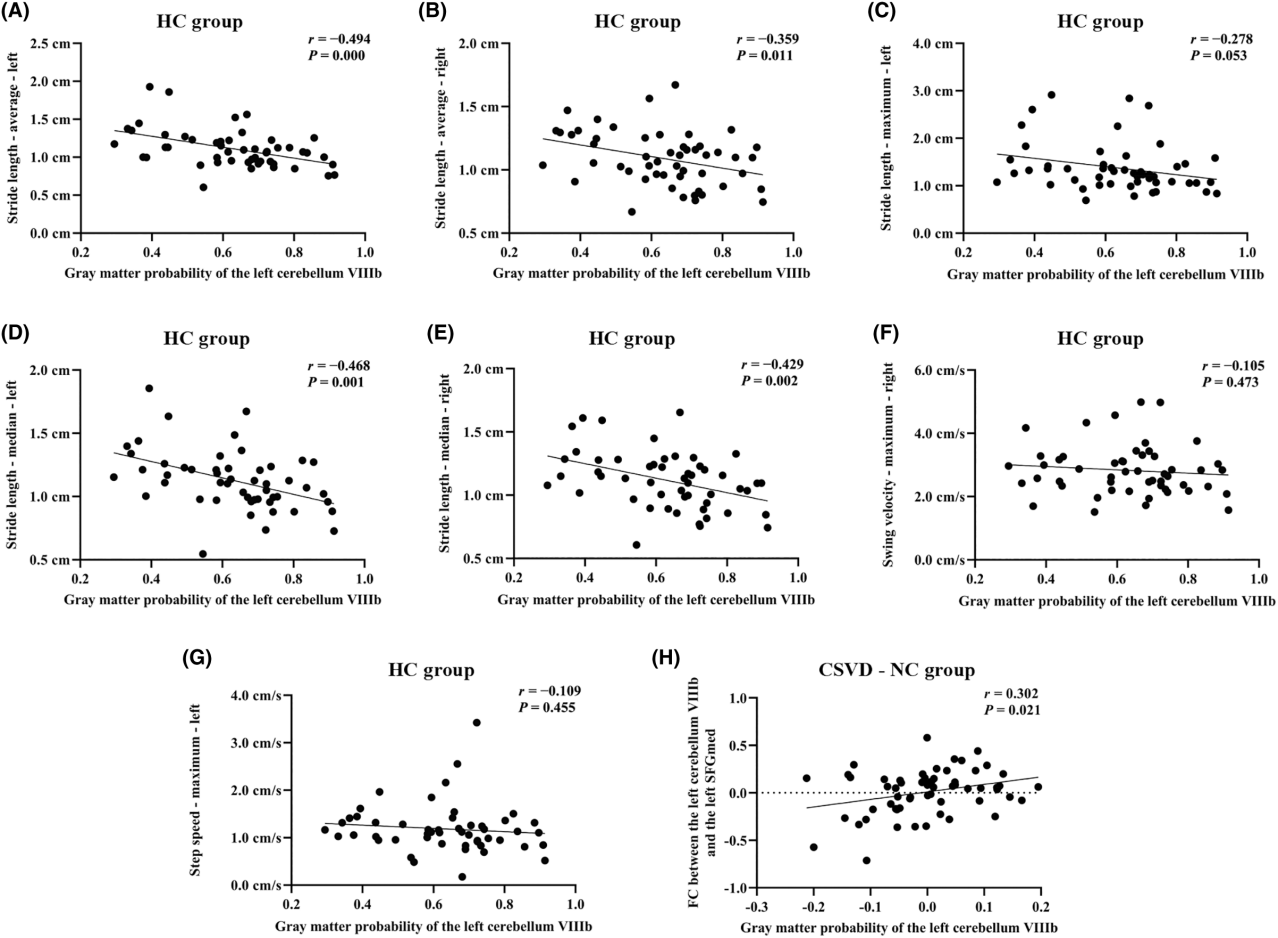
Significant correlations in the HC and CSVD‐NC groups. (A–G) Significant correlations between gait indicators and neuroimaging parameters in the HC group. The correlation analyses between gait indicators and neuroimaging parameters were performed by partial correlation, adding age, white matter hyperintensity volume, and body mass index as covariates. (H) Positive correlation between the gray matter probability of the left cerebellum VIIIb and the functional connectivity between the left cerebellum VIIIb and the left SFGmed in CSVD‐NC group, adjusted for age and white matter hyperintensity volume. CSVD‐NC, patients with cerebral small vessel disease with normal cognitive function; FC, functional connectivity; HC, healthy controls.

We first focused on the relationship between gait indicators and neuroimaging parameters. In the HC group, the gray matter probability of the left cerebellum VIIIb was negatively related to stride length‐average‐left (*r* = −0.494, *p* = 0.000), as well as stride length‐average‐right (*r* = −0.359, *p* = 0.011), stride length‐median‐left (*r* = −0.468, *p* = 0.001), and stride length‐median‐right (*r* = −0.429, *p* = 0.002), adding age, white matter hyperintensity volume, and body mass index as covariates. Notably, there was no significant correlation between gait indicators and neuroimaging parameters in the CSVD‐NC and CSVD‐MCI groups. Significantly, the gray matter probability of the left cerebellum VIIIb was positively correlated to the FC between the left cerebellum VIIIb and the left SFGmed (*r* = 0.302, *p* = 0.021) only in CSVD‐NC group, adjusted for age and white matter hyperintensity volume. Total CMBs, total lacunes, and WMH volume were not correlated to gait indicators in either the CSVD‐NC or CSVD‐MCI groups (Figure 5).

The correlation between gait indicators and neuropsychological data was subsequently explored. The gait indicators indicated a remarkable association with DST, CVF, SCWT interference, TMT, BNT, AVLT‐short time delay recall, and AVLT‐long time delay recall in the CSVD‐MCI group. Detailed information is shown in Table 5.

**TABLE 5:** Significant correlation between gait indicators and neuropsychological data in the CSVD‐MCI group.

		Stride length‐average‐left	Stride length‐average‐right	Stride length‐median‐left	Stride length‐median‐right	Stride length‐maximum‐left	Step speed‐maximum‐left	Swing velocity‐maximum‐right
DSF^a^	Correlation coefficient		0.349	0.340	0.356		0.307	0.407
	*p‐*value		0.010	0.012	0.008		0.024	0.002
DSB^a^	Correlation coefficient	0.444	0.364	0.459	0.431	0.415	0.411	0.500
	*p‐*value	0.001	0.007	0.000	0.001	0.002	0.002	0.000
CVF^b^	Correlation coefficient	0.334	0.311	0.345	0.311	0.325	0.322	
	*p‐*value	0.016	0.025	0.012	0.025	0.019	0.020	
SCWT‐interference^b^	Correlation coefficient	−0.413	−0.317	−0.428	−0.376			
	*p‐*value	0.002	0.020	0.001	0.005			
TMT‐A^a^	Correlation coefficient	−0.335	−0.356	−0.350	−0.370	−0.318	−0.373	−0.417
	*p‐*value	0.011	0.007	0.008	0.005	0.016	0.004	0.001
TMT‐B^b^	Correlation coefficient		−0.329		−0.304			
	*p‐*value		0.029		0.045			
AVLTSTDR^b^	Correlation coefficient			0.333	0.321			
	*p‐*value			0.013	0.017			
AVLTLTDR^a^	Correlation coefficient	0.335		0.370	0.303			0.300
	*p‐*value	0.012		0.005	0.024			0.026
BNT^a^	Correlation coefficient						0.304	0.335
	*p‐*value						0.027	0.014

Abbreviations: AVLTLTDR: Auditory Verbal Learning Test‐long time delay recall; AVLTSTDR: Auditory Verbal Learning Test‐short time delay recall; BNT, Boston Naming Test; CSVD‐MCI, patients with cerebral small vessel disease with mild cognitive impairment; CVF, Category Verbal Fluency; DSB, Digit Span Test‐backward; DSF, Digit Span Test‐forward; SCWT, Stroop Color Word Test; TMT, Trial Making Test.

^a^ Analyzed by Spearman correlation.

^b^ Analyzed by partial correlation, adding age, white matter hyperintensity volume, body mass index, and education as covariates.

In addition, the correlation between neuroimaging parameters and neuropsychological data was also computed. Only BNT significantly correlated with the FC between the right STG and left IOG (*r* = −0.366, *p* = 0.009), as well as the FC between the right STG and left ITG (*r* = −0.325, *p* = 0.021) in the CSVD‐MCI group.

## 4 DISCUSSION

This cross‐sectional study determined the gait and neuroimaging patterns in patients with CSVD with and without MCI, focusing on the relationship among gait, cognition, and cerebellar neuroimaging features. The results mainly revealed that (1) GD existed in patients with CSVD regardless of whether there was CI or not; (2) the performance of stride length, step speed, and swing velocity deteriorated as cognitive function decreased in patients with CSVD compared with HCs, and the stride length may be typical for early GD in CSVD; (3) the structural atrophy and functional downregulation of the left cerebellum VIIIb may be responsible for early GD in patients with CSVD; and (4) linguistic function, memory, and attention were closely related to gait performance in patients with CSVD with MCI.

We first report that the stride length was reduced in patients with CSVD without CI and decreased gradually with cognitive decline in CSVD, which indicated stride length might be the earliest characteristic of GD in CSVD. The results suggest that gait was already deficient in patients with CSVD before MCI. However, the causal relationship between early GD and CI in patients with CSVD cannot be established, which is similar to the results from Vyara's research.^25^ Karlijn et al. reported stride length was the most sensitive parameter related to white matter lesions.^26^ In another American study, there were also differences in stride length between patients with CSVD and controls.^27^ Different from previous studies of CSVD, we did not find a significant difference in gait velocity between HCs and patients with CSVD.^28^, ^29^, ^30^ Actually, in previous studies of gait in CSVD, seldom were subjects with dementia excluded, resulting in a potential influence on the results. Besides, step speed and swing velocity were also decreased in patients with CSVD with MCI compared with HCs, which needs to be further verified by expanding the sample size.

The patterns of GD vary among different diseases. Poor gait in CSVD has been shown to be more strongly associated with vascular dementia rather than AD.^31^ In an American study of 1719 participants, spatiotemporal gait parameters were more disturbed in people with non‐AD dementia than in those with AD.^32^ In studies of aging, a decline in gait velocity is a common indicator of abnormality.^33^ In a study of MCI and AD, it was also gait velocity rather than stride length that was reported to decline before CI.^6^, ^7^ Overall, gait indicators may be useful for distinguishing between different neurological diseases. Admittedly, the centralized reporting on gait velocity might be the result of limited evaluation techniques of gait in previous studies. The gait testing system we used calculated gait indicators using artificial intelligence technology, from the perspective of time, space, and space–time and may provide a useful tool for further exploring the gait characteristics of different diseases in the future.

Normal gait control is a complex function that depends on the coordination of multiple brain regions, including the cerebellum.^34^ We found that an alteration in the cerebellum VIII may play a crucial role in GD in CSVD, extending the findings of our previous study.^5^ Our results suggest that the cerebellum might be more important for GD in CSVD than the cerebrum. Studies have found the observed pattern of cerebellar lobular atrophy was related to motor and cognitive ability scores.^35^ Cerebellum VIII, a sensorimotor region,^36^ is associated with gait, balance, motor performance and has feedforward and feedback links with the frontal motor cortex.^8^ In this study, the gray matter probability of the left cerebellum VIIIb in patients with CSVD was lower than in HCs and decreased with cognitive decline. What is more, it was the gray matter probability of the left cerebellum VIIIb rather than traditional CSVD neuroimaging features (CMBs, total lacunes, or WMH volume) that was negatively related to stride length in the HC group, which disappeared with the occurrence of CSVD. Besides, accompanied by atrophy of the left cerebellum VIIIb, it was also observed that the FC between the left cerebellum VIIIb and left SFGmed was significantly reduced in patients with CSVD compared with HCs. In other words, the left cerebellum VIIIb was involved in early GD in patients with CSVD, not only structurally but also functionally. Frontal regions and deep gray matter atrophy were observed in elderly fallers as well as alterations in FC within large‐scale brain networks.^37^ SFGmed is considered an information receiver and processor and is responsible for muscular control, especially in fine motor functions. Abnormal modulation of neuronal activity or brain network attributes in SFGmed was observed in patients with motor dysfunction.^38^ In a time‐course study with fMRI, activity in the SFGmed and posterolateral cerebellum were found to be engaged in the willed generation of virtual motor commands and analysis of virtual sensory signals.^39^ Notably, many researchers considered that GD and impaired executive function in CSVD were closely related. However, there was no significant association between executive function and gait indicators with intergroup differences in our study, which is worth exploring in the future.

Previous studies suggested that there was a correlation between gait slowing and CI, which was related to the support of a common neural base.^40^ We also found that stride length gradually decreased with the occurrence of CI. Moreover, the gait indicators were observed to be associated with DST, CVF, SCWT interference, TMT, BNT, and AVLT, which are involved in linguistic function, memory, and attention, in patients with CSVD with MCI. The CSVD‐MCI group had a thinner cortex in the left fusiform, left rectus, and right STG compared to the HC and CSVD‐NC groups. Previous research has reported cortical atrophy, including in the frontal cortex, bilateral calcarine sulcus, and fusiform gyrus, in vascular CI caused by CSVD.^41^ Although our results cannot support a causal relationship between GD and CI in CSVD, CI was indeed related to the worsening of GD, which may be owing to the sharing of brain functional areas between cognition and gait. The reason for occurrence and deterioration of CSVD has always been a research focus. The interaction points between gait and cognition may have important implications for research into the mechanisms underlying CSVD. We also found that BNT significantly correlated to the FC between the right STG and left IOG, as well as the FC between the right STG and left ITG in the CSVD‐MCI group, which may provide some reference for studying the brain area responsible for linguistic impairment in patients with CSVD.

The limitations of this study are as follows. First, the sample size was not very large, and study groups differed in terms of age. Accounting for this, we adjusted for age in our statistical analyses. Second, in this cross‐sectional study, several gait indicators showed significant differences between the patients with CSVD and HCs, while there was only a trend rather than a statistical difference between the CSVD‐NC and the CSVD‐MCI groups. In the future, we will enroll more subjects to verify these findings and construct machine‐learning models to demonstrate the core role of gait indicators in predicting the occurrence of CSVD and associated cognitive deterioration. Notably, the aberrations of structural and functional MRI in this study showed a mismatch, which also deserves to be further explored in the future.

In conclusion, GD appears before CI in CSVD. Decreased stride length was the earliest characteristic of GD in CSVD. The structural and functional regulation of the left cerebellum VIIIb could play a particularly important role in early GD of CSVD.

## AUTHOR CONTRIBUTIONS

YX: conceptualization. YM, BJ, ZK, CM, JJ, RQ, DY, ZH: investigation. YM, BJ, ZK, CM, LH, and YH: formal analysis. YM and BJ: writing—original draft. YM, BJ, and YX: writing—review and editing. All authors contributed to the article and approved the submitted version.

## FUNDING INFORMATION

This research was supported by the National Science and Technology Innovation 2030—Major program of “Brain Science and Brain‐Like Research” (2022ZD0211800), the Key Research and Development Program of Jiangsu Province of China (BE2020620), Jiangsu Province Key Medical Discipline (ZDXKA2016020), and the National Natural Science Foundation of China (81920108017, 82130036).

## CONFLICT OF INTEREST STATEMENT

Xu, Yun is an Editorial Board member of CNS Neuroscience and Therapeutics and a co‐author of this article. To minimize bias, they were excluded from all editorial decision‐making related to the acceptance of this article for publication.

## Supporting information


Appendix S1.

## Data Availability

All supporting data contributing to this study can be provided from the corresponding author on reasonable request.
