# Supplementary material for: Stride length and cerebellar regulation: Key features of early gait disorder in cerebral small vessel disease
Source: CNS Neurosci Ther. 2024 Feb 7;30(2):e14545. doi: 10.1111/cns.14545 (PMC10850804; doi:10.1111/cns.14545)
Supplement: Supplementary file 1 — Appendix S1. [file CNS-30-e14545-s001.doc]

Supplementary Material

**Stride length and cerebellar regulation: key features of early gait disorder in cerebral small vessel disease**

**Yuting Mo ^a, #^, Biying Ji ^a, #^, Zhihong Ke ^a^, Chenglu Mao ^b, e^, Jialiu Jiang ^b, e^, Yanan Huang ^b, e^, Ruomeng Qin ^b, e^, Lili Huang ^b, e^, Dan Yang ^a^, Zheqi Hu ^b, e^, Yun Xu ^a, b, c, d, e, *^**

^#^ These authors contributed equally to this work.

*Corresponding author at: Department of Neurology, Nanjing Drum Tower Hospital, Clinical College of Nanjing Medical University, Nanjing 210008, China. E-mail address: xuyun20042001@aliyun.com (Yun Xu).

1. **Methods**
   1. Demographic data collection and neuropsychological evaluation

The demographic data collection included sex, age, education, body mass index (BMI), and vascular risk factors. The standardized neuropsychological test protocol included Hamilton Depression Rating Scale (HAMD)^1^ and Hamilton Anxiety Rating Scale (HAMA),^2^ which evaluated the mental statements, as well as Minimum Mental State Examination (MMSE), Montreal Cognitive Assessment (MoCA)^3^ (Beijing version 26 August 2006 translated by Wei Wang & Hengge Xie, www.mocatest.org), Auditory Verbal Learning Test (AVLT),^4, 5^ Digit Span Test (DST), Category Verbal Fluency (CVF), Wechsler Memory Scale-Visual Reproduction (VR), Stroop Color Word Test (SCWT), Trial Making Test (TMT), Auditory Verbal Learning Test (AVLT), Boston Naming Test (BNT), and Visual Objective and Space Perception Battery - silhouettes (VOSP - silhouettes).

Global cognition was assessed by MMSE and MoCA. The education-adjusted cut-off values for the MMSE were > 17 for illiteracy, > 20 for 1 ‒ 6 years of education, and > 24 for > 6 years of education. The education-adjusted cut-off values for the MoCA were > 13 for illiteracy, > 19 for 1‒6 years of education, > 24 for 7‒12 years of education, and ≥ 26 for > 12 years of education. DST, CVF, VR, SCWT, TMT, AVLT, BNT, and VOSP - silhouettes were used to conduct a detailed cognitive assessment involving multiple cognitive domains. AVLT is a classic test for verbal memory and contains four main indicators: AVLT - immediate recall (AVLTIR), AVLT - short term delayed recall (AVLTSTDR), AVLT - long term delayed recall (AVLTLTDR), and AVLT - recognition (AVLT - recognition). To make the memory evaluation more comprehensive, the VR was also conducted for the assessment of visuospatial memory and the scores of VR - immediate recall (VRIR), VR - delayed recall (VRDR), and VR - recognition (VRR), were statistically analyzed. DST is a simple method for memory and attention. There are two parts to the DST: DST - forward (DSF) and DST-backward (DSB). Linguistic function was evaluated by CVF and BNT. SCWT, which includes SCWT - dot, SCWT - word, SCWT - interference, SCWT - Interference - Dot (SCWT - interference minus SCWT - dot), and SCWT - Interference - Word (SCWT - interference minus SCWT - word), could access processing speed and executive function. TMT, which includes TMT - A, TMT - B, TMT - BA (TMT - A minus TMT - B), was used to evaluate executive function and space perception. Complementally, VOSP - silhouettes was also conducted to access space perception and visual objective.

- 1. Magnetic Resonance Imaging (MRI) data acquirement

The MRI scanning was conducted in the Nanjing Drum Tower Hospital with a Philips 3.0-T scanner (Philips Medical Systems, Netherlands). The multimodal MRI scans included 1) the high-resolution T1-weighted turbo gradient echo sequence, with repetition time (TR) = 9.8 ms, flip angle (FA) = 8^◦^, echo time (TE) = 4.6 ms, field of view (FOV) = 250 mm × 250 mm, number of slices = 192, acquisition matrix = 256 × 256, thickness = 1.0 mm, 2) the fluid attenuated inversion recovery (FLAIR) sequence, with TR = 4,500 ms, TE = 333 ms, time interval (TI) = 1,600 ms, number of slices = 200, voxel size = 0.95 mm × 0.95 mm × 0.95 mm, acquisition matrix = 270 × 260, and 3) the blood oxygen on level depending (BOLD) sequence, TR = 2000ms, TE = 30 ms, number of slices = 35, voxel size = 3.00 mm × 3.00 mm × 4.00 mm, acquisition matrix = 64 × 62.

- 1. MRI data analysis
     1. Structural MRI data
        1. Quantification of white matter hyperintensity (WMH) and total intracranial volume (TIV)

The lesions of white matter were quantified by Lesion Segmentation Tool (LST),^6^ which is a toolbox based on statistical parametric mapping software package (SPM; [www.fil.ion.ucl.ac.uk/spm)](http://www.fil.ion.ucl.ac.uk/spm)) 12, based on T2-weighted FLAIR sequence and T1-weighted sequence. Detailed parameters are as follows: initial threshold: 0.15, parameter for the Markov Random Field: 1, maximum iterations: 50. The result of LST was defined as the value of WMH volume. Voxel-based morphometry toolbox for SPM8 (VBM8), a toolbox based on SPM8, was used to quantify the volume of gray matter, white matter, and cerebrospinal fluid. TIV was the sum of the volume of gray matter, white matter, and cerebrospinal fluid.

1.3.1.2 Cerebral structure analysis

FreeSurfer (version 7.2 for Linux)^7^ was used to perform the analysis of cerebral cortex structure, based on the high-resolution T1-weighted turbo gradient echo sequence. The pre-processing steps were as follows: 1) motion correction and conform; 2) non-uniform intensity normalization; 3) Talairach transformation; 4) intensity normalization;^8^ 5) stripping skull; 6) automatic subcortical segmentation of the white matter and the deep gray matter volumetric structures; 7) segmentation of the brain tissue, included gray matter (GM), white matter (WM), and cerebrospinal fluid (CSF); 8) automated topology fixer; 9) surface modeling for the GM/WM and GM/CSF boundaries;^9, 10^ 10) surface inflation;^11^ 11) registration to average surface space;^12^ 12) smoothing with a 10-mm full-width half-maximum (FWHM) Gaussian spatial kernel for noise reduction. The cortical thickness was calculated as the closest distance from the inner surface to the outer surface at each vertex.^13^ The Desikan - Killiany (DK) atlas was chosen for consistent brain measurements,^14^ as the boundaries in it are suitable for the FreeSurfer classifier.

Glmfit (analytic software in FreeSurfer) was used to analyze the whole-brain vertex-wise surface-based cortical thickness. A general linear model (GLM) was built by a different offset same slop method. Because of the participants’ diversity demographic characteristics, age and TIV were served as the covariances for the GLM analysis. Multiple comparison corrections were performed using Monte Carlo simulation correction a vertex-wise/ cluster-forming threshold of 3 (P < 0.001) and a cluster-wise P < 0.05. The cortical thickness of significant brain regions was extracted for further analysis.

1.3.1.3 Cerebellar structure analysis

A spatially unbiased atlas template of the cerebellum and brainstem (SUIT),^15^ a toolbox based on SPM12, was applied to the analysis of cerebellar gray matter structure, based on the high-resolution T1-weighted turbo gradient echo sequence. The detailed processing steps were as follows: 1) cerebellar isolation and segmentation; 2) normalizing an individual cerebellum into the SUIT atlas template by Diffeomorphic Anatomical Registration Through Exponentiated Lie algebra (DARTEL); 3) reslicing images into SUIT space using DARTEL; 4) smoothing images with a 3-mm FWHM Gaussian spatial kernel. The gray matter probability of cerebellar region showing significant difference in two sample t-test (covariance: age; multiple comparison corrections: Gaussian random field [GRF]-corrected threshold of p < 0.001 at the voxel level and p < 0.05 at the cluster level) was extracted for further analysis by Data Processing Assistant for Resting-State Functional MR Imaging toolkit (DPARSF)^16^.

- - 1. Functional MRI (fMRI) data

The fMRI analysis was based on the BOLD sequence. DPARSF, a method based on the Resting-State Functional MR Imaging Toolkit (http://www.restfmri.net) and SPM12, was used for fMRI data analysis. The preprocessing steps included removing the first ten volumes of data, slice timing correction, realignment (note that the subjects with head motion more than 3.0 mm of displacement in any direction, or 3.0 degrees of rotation in any angular dimension were excluded), reorientation, co-registration of T1 images to functional images; image segmentation for the high-resolution T1-weighted turbo gradient echo sequence and the BOLD sequence by DARTEL, nuisance covariates regression (including linear detrending, Friston 24 head motion parameters, white matter signal, cerebrospinal fluid signal, and global signal), normalization using a 12- parameter nonlinear transformation to the standard Montreal Neurological Institute (MNI) space (3 × 3 × 3 mm^3^), smoothing functional images with a 6-mm FWHM Gaussian spatial kernel, and band-pass filtering (0.01-0.08Hz).

The MNI coordinates of peak vertex in the left fusiform, left rectus, right STG, as well as the MNI coordinate of peak voxel in the left cerebellum VIIIb were defined as the spherical center of regions of interest (ROIs) (5-mm radius spheres) in FC analysis. The mean time series of the ROI was extracted, and the strengths of FC were calculated as Pearson's correlations between the averaged time series of the ROI and voxels in the remaining brain regions. Then, Pearson's correlation coefficients were normalized to a z-score matrix by using a Fisher r-to-z transformation, and the FC map was created between the ROI and the remaining brain regions. Analysis of covariance (ANCOVA) of FC among the HC, CSVD - NC, and CSVD - MCI groups was implemented by DPARSF (multiple comparison corrections: GRF-corrected threshold of p < 0.001 at the voxel level and p < 0.05 at the cluster level), adjusted for age. The FC values of significant brain regions were extracted for correlation analysis.

**References**

1. Hamilton M. A rating scale for depression. J Neurol Neurosurg Psychiatry. 1960;23(1):56-62.

2. Hamilton M. The assessment of anxiety states by rating. Br J Med Psychol. 1959;32(1):50-5.

3. Lu J, Li D, Li F, Zhou A, Wang F, Zuo X, et al. Montreal cognitive assessment in detecting cognitive impairment in Chinese elderly individuals: a population-based study. J Geriatr Psychiatry Neurol. 2011;24(4):184-90.

4. Zhao Q, Lv Y, Zhou Y, Hong Z, Guo Q. Short-term delayed recall of auditory verbal learning test is equivalent to long-term delayed recall for identifying amnestic mild cognitive impairment. PLoS One. 2012;7(12):e51157.

5. Zhao Q, Guo Q, Liang X, Chen M, Zhou Y, Ding D, et al. Auditory Verbal Learning Test is Superior to Rey-Osterrieth Complex Figure Memory for Predicting Mild Cognitive Impairment to Alzheimer's Disease. Curr Alzheimer Res. 2015;12(6):520-6.

6. Schmidt P, Gaser C, Arsic M, Buck D, Forschler A, Berthele A, et al. An automated tool for detection of FLAIR-hyperintense white-matter lesions in Multiple Sclerosis. Neuroimage. 2012;59(4):3774-83.

7. Fischl B. FreeSurfer. Neuroimage. 2012;62(2):774-81.

8. Sled JG, Zijdenbos AP, Evans AC. A nonparametric method for automatic correction of intensity nonuniformity in MRI data. IEEE Trans Med Imaging. 1998;17(1):87-97.

9. Dale AM, Fischl B, Sereno MI. Cortical surface-based analysis. I. Segmentation and surface reconstruction. Neuroimage. 1999;9(2):179-94.

10. Fischl B, Liu A, Dale AM. Automated manifold surgery: constructing geometrically accurate and topologically correct models of the human cerebral cortex. IEEE Trans Med Imaging. 2001;20(1):70-80.

11. Fischl B, Sereno MI, Dale AM. Cortical surface-based analysis. II: Inflation, flattening, and a surface-based coordinate system. Neuroimage. 1999;9(2):195-207.

12. Fischl B, Sereno MI, Tootell RB, Dale AM. High-resolution intersubject averaging and a coordinate system for the cortical surface. Hum Brain Mapp. 1999;8(4):272-84.

13. Fischl B, Dale AM. Measuring the thickness of the human cerebral cortex from magnetic resonance images. Proc Natl Acad Sci U S A. 2000;97(20):11050-5.

14. Klein A, Tourville J. 101 labeled brain images and a consistent human cortical labeling protocol. Front Neurosci. 2012;6:171.

15. Diedrichsen J. A spatially unbiased atlas template of the human cerebellum. Neuroimage. 2006;33(1):127-38.

16. Chao-Gan Y, Yu-Feng Z. DPARSF: A MATLAB Toolbox for "Pipeline" Data Analysis of Resting-State fMRI. Front Syst Neurosci. 2010;4:13.
